# Enhanced synthesis of andrographolide by *Aspergillus niger* and *Penicillium expansum* elicitors in cell suspension culture of *Andrographis paniculata* (Burm. f.) Nees

**DOI:** 10.1186/1999-3110-54-49

**Published:** 2013-10-24

**Authors:** Moinuddin M A Vakil, Vijay D Mendhulkar

**Affiliations:** Department of Botany, The Institute of Science, 15, Madam Cama Road, Mumbai, 4000 32 India

**Keywords:** Active constituents, Fungal elicitors, HPLC analysis, Secondary metabolites, Tissue culture techniques

## Abstract

**Background:**

*Andrographis paniculata* (Burm. f.) Nees is an important medicinal plant which has enormous applications in pharmaceutical industries. Cell suspension culture of *Andrographis paniculata* (Burm. f.) Nees. was treated with *Aspergillus niger* and *Penicillium expansum* elicitors to enhance the synthesis of andrographolide, the bioactive constituent of *A. paniculata*.

**Result:**

The elicitation treatment with fungal elicitors (*A. niger and P. expansum*) was observed to be most suitable for eliciting andrographolide production in the culture. The quantification of andrographolide was done using High Performance Liquid Chromatography (HPLC) technique. *A. niger* extract (1.5 ml with10 days treatment duration) revealed 6.94 fold increase in andrographolide content (132 μg) which was higher than the control (19 μg). *P. expansum* elicitor (0.6% with 8 days treatment duration) could reveal 6.23 fold enhancement in andrographolide content (81.0 μg) over control (13 μg).

**Conclusion:**

The results obtained reveal that the longer treatment duration is most favorable for the elicitation of andrographolide using both the fungal elicitors.

**Electronic supplementary material:**

The online version of this article (doi:10.1186/1999-3110-54-49) contains supplementary material, which is available to authorized users.

## Background

Plant cell culture is considered as a useful technique for the large scale production of plant secondary metabolites under in vitro level beside its utility for micropropagation. The production of these bioactive metabolites can be achieved by elicitation, cell immobilization, permeabilization (Brodelius et al.^1989^; Mendhulkar et al.^2009^), alteration in media composition etc.

*Andrographis paniculata* Nees. is commonly known as ‘Kalmegh’ in India and as a medicinal plant belongs to the family Acanthaceae. The plant is recommended for its drug utility in Indian Pharmacopoeia and widely used in Ayurveda, Unani, Siddha and Homeopathy systems of medicines. The plant is reported to possess terpenoids and flavonoids. The major terpenoids viz. 14-deoxy-11-oxoandrographolide, 14-deoxy-11, 12-didehydroandrographolide and 14-deoxyandrographolide andrographolide are the active constituents of this plant. The main active constituent is andrographolide which is reported to possess liver stimulant, astringent, anodyne, tonic and alexipharmic properties and useful in dysentery, cholera, diabetes, consumption, influenza, bronchitis, swellings, itches, piles and gonorrhea (Zhao and Frang^1991^). The most significant pharmaceutical properties of this plant are anticancerous (Kumar et al.^2004^) and anti-HIV (Calabrese et al.^2000^).

The qualitative and quantitative improvement of biologically active compounds by plant cell, tissue and organ culture have been used as an alternative source for the production of important bioactive secondary metabolites using different strategies of metabolic engineering. The biotechnological production of valuable secondary metabolites in plant cell or organ cultures is a good alternative to the extraction of whole plant material because many plants of high medicinal products are difficult to cultivate. The attractive concepts in the scaling up technology of natural products are enhancement of secondary metabolites and use of biological and chemical elicitors. It has been found that plants elicit the same response when come in contact with the compounds of the pathogen as attacked by the pathogen itself. These compounds are known as elicitors. Taxol, an anticancer drug produced from cell cultures of *Taxus* plant has been successfully industrialized for commercial purpose. Ajmalicine and serpentine from *Catharanthus roseus,* Caffeine from *Coffea Arabica,* Ginsenoside from *Panaxginseg* and Vomilenine from *Rauwolfia serpentine* have been produced from cell cultures (Bojwani and Razdan^1996^). It has also been successfully experimented for the compounds like Cardio active glycosides in *Digitalis purpurea*, L-Dopa in *Mucuna prurience*, Berberine in *Coptis japonica*, diosgenin in *Dioscorea deltoid* and capsaicin in *Capsicum frutescent* (Masanaru^1994^; Vanisree et al.^2004^).

According to WHO, Angiospermic plants contribute 11% of 252 drugs which are considered as basic and essential drugs (Rates^2001^). Plants derived drugs in western countries have a huge market value. Prescription drugs containing photochemical were valued at more than US$30 billion in 2002 in the USA alone (Raskin, et al.^2002^). The average yield of these plants are about 2.5 tonnes/ ha and it is sold in Indian market at the rate of Rs.15-20 / Kg. (Purohit and Vyas^2004^). In Indian market, the rate of *A. paniculata* crude powder is Rs. 1800/- per quintal. This powder is used in Ayurvedic and Homeopathic formulations.

*Andrographis paniculata* (Kalmegh) forms the part of number of drug formulations available in Indian market. It is a major constituent of SG-1 Switradilepa which is an Ayurvedic drug effective in treating vitiligo -a dermatological disease. It is also used in certain homoeopathic preparations such as Livo −10 (syrup) which is used in various functional derangements of liver and gastro intestinal system, acute and chronic hepatitis, poor digestion, constipation and Paroxysmal pain in abdomen. It is one of the important constituents of herbal formulations, Purim, a blood purifier (capsule and syrup); Liv fit, a hepatitis virus DNA replication inhibitor drug and a multiple disorder curetting drug, Livo Plus. All these drugs are available in Indian market.

In the present work, elicitation of andrographolide compound was experimentally worked out using two fungal elicitors viz. *Aspergillus niger* and *Penicillium expansum* in *Andrographis paniculata* (Burm.f.) Nees. cell suspension culture.

## Methods

### Cell suspension culture

**Figure 1:**
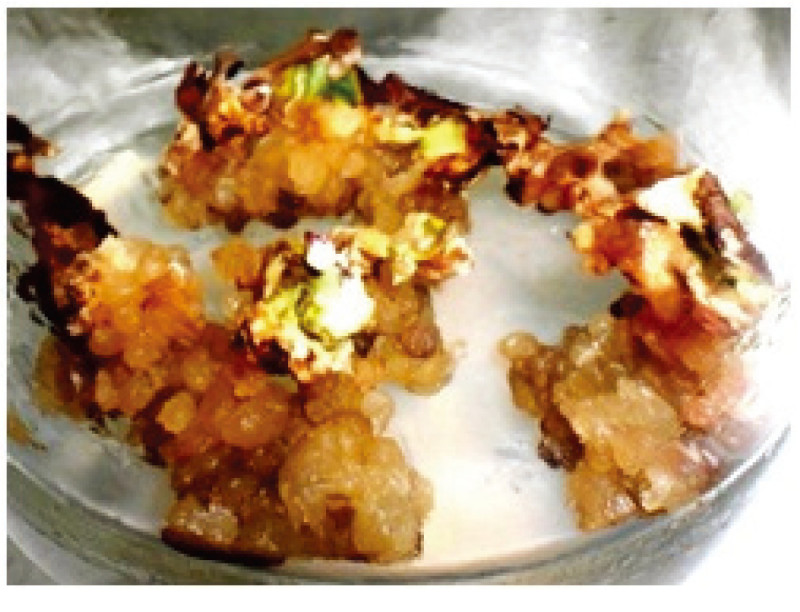
***A. paniculata*** **callus.**

The calli obtained using leaf explants of *Andrographis paniculata* on MS media supplemented with 2,4-D: BAP (1.0:0.5 mg/l) were transferred into liquid Murashige and Skoog^1962^media supplemented with same hormonal combination and concentration except agar (Figure 1). The cell suspension culture was grown in gyratory shaker with 120 r.p.m. at 25 ± 1°C under dark condition for 30 days. The cell growth phase was determined using colorimetric method.

### Cell viability

The cell viability in 25 days old suspension culture was determined by Guava Via Count assay using Guava Easy CD4 System. For cell viability assay, 10 μl of suspension culture was taken in eppendorf tube, added 190 μl of via count solution (viability counting solution) and incubated for 5 min at room temperature. The analysis was performed using Cytosoft software for Guava Via Count (Guava ViaCount Reagent^2006^). The results were obtained in the form of viable cell count and total cell count (both in number of cells/ml) and the percent viability.

### Fungal culture

*Aspergillus niger* and *Penicillium expansum* used for elicitation were obtained from Department of Microbiology (The Institute of Science, Mumbai). The fungal cultures were first maintained separately as slants in culture tubes containing Potato dextrose agar (PDA) medium. The culture medium composition is as follows in g/L: Peeled potatoes (200 g), Dextrose (20 g), Yeast extract (0.1 g), Agar (20 g), Twin 80 (0.5%) and pH was adjusted to 5. After two weeks, both the fungi were grown separately in 250 ml flasks containing 100 ml of Potato dextrose broth (PDB medium) under dark and static condition at room temperature for three weeks. PDB was prepared similarly without agar which was used to grow fungal mycelia for the preparation of elicitor.

### Preparation of *Aspergillus niger* elicitor

*Aspergillus niger* cultures grown in 250 ml flasks containing 100 ml of potato-dextrose broth (PDB medium) were harvested after three weeks. The fungal cultures were autoclaved along with the media at 15 psi for 20 min. After autoclaving, the fungal mycelial mat floating on the surface of the medium was removed carefully and washed several times with distilled water and allowed to dry in hot air oven at 40°C to obtain constant dry weight. The dried mycelial mat was crushed into powder using mortar and pestle and used as dry cell powder (DCP).

### Extraction of DCP

Five g of DCP was added to 500 ml of acidified distilled water (pH 2) and boiled for 45 min. After boiling, the culture was filtered and the pH of the filtrate was adjusted to 5 using 1 N NaOH and the volume was adjusted to 500 ml with D.W. This solution was autoclaved at 15 psi for 20 min. and used as an elicitor without further purification. 1 ml, 1.5 ml and 2 ml of this fungal elicitor were added in Dippy’s Jar containing the *A. paniculata* cell suspension. The treatments of fungal elicitor were administered for the duration of 4 days, 7 days and 10 days.

### Preparation of *Penicillium expansum* elicitor

*Penicillium expansum* mycelial mat obtained after three weeks was autoclaved, separated and washed several times with demineralized water. The mycelial residue was resuspended in an amount of demineralized water equal to that of the filtrate and homogenized. This homogenate was autoclaved again and used in the concentrations, 0.3%, 0.6% and 1.2%. The treatment was administered in Dippy’s Jar containing *Andrographis paniculata* cell suspension. Each of these fungal concentrations was subjected for the treatment duration of 2 days, 5 days and 8 days. The polysaccharide content in the DCP extract *of Aspergillus niger* and *Penicillium expansum* homogenate was determined by the phenol sulfuric acid method (Sadasivam and Manickam^2005^) using glucose as the standard.

### Analytical methods

After each experiment, cell suspensions were filtered and washed several times with distilled water and filtered cells were dried in oven at 50°C to a constant weight. Completely oven dried samples of all treatments were powdered using mortar and pestle. Amount of 150 mg of was taken from each powdered sample was and sonicated using 2 mm probe for 10 min with pulse rate operating at 10 sec. on and 2 sec. off, amplitude 20% in 1.5 ml of methanol using Sonics Vibra Cell (VCX 130) instrument. After sonication the extract was centrifuged at 5000 rpm for 5 min. the supernatant was transferred into 2 ml eppendorf tubes and the final volume was adjusted to 2 ml with methanol (HPLC grade). This extract was used for HPLC analysis. For HPLC analysis, 2 ml of methanol extract was filtered with 0.45 mm nylon syringe filter (Tarsons). The filtered extracts were stored in clean HPLC vials before injection into HPLC system.

Andrographolide content in the samples was detected and quantified by HPLC system, supplied by Agilent Technologies comprising 1100/1200 Column Thermostat, 1200 Variable Wavelength Detector, 1100/1200 Quaternary Pump and 100/1200 Thermostatted Autosampler. Column of ZORBAX 300SB-C18, dimensions 4.6 × 150 mm (“Agilent Technologies”, USA) with particle size of 5 μm were used. The column was operated in the reverse phase mode. The compound, andrographolide in the samples was detected at 223 nm wavelength. All the solvents used were of HPLC grade (“Fisher Scientific”, India and Sigma - Aldrich). The purified RO water (Analytica, India) was used for preparing mobile phase. All the samples were filtered through 0.45 mm membrane filter. The mobile phase consisted of a mixture of Acetonitrile: 0.1% (v/v) phosphoric acid in water (40: 60, v/v) under isocratic conditions for analysis of andrographolide. 20 μl of each sample was used for injection and the flow rate was 1.0 ml/min. and the run was continued for 8 minutes for column 4.6 × 150 mm for all samples for complete resolution and detection of andrographolide. The 20 ppm standard solution of andrographolide was prepared in HPLC grade methanol and 20 μl from 20 ppm solution was used for injection. Injection volume of 20 μl was kept constant during the experimentation. Identification of andrographolide in cell samples was confirmed by retention time, co-chromatography with the standard and peak purity by wavelength.

### Statistical analysis of data

All the data obtained is represented as mean ± SD of triplicate of experiments using Microsoft Excel XP.

## Results and discussion

### Cell viability

The cell viability of cell suspension culture of *Andrographis paniculata* was recorded to be 86% using Guava Easy CD4 System (Table 1). This result indicates that the conditions for cell suspension culture are quite favorable. The same incubation condition was applied for further experimental cultures.

**Table 1:** **Cell viability in** ***Andrographis paniculata*** **suspension culture after 30 days incubation in gyratory shaker at 110 rpm at 25 + 2°C**

Sample ID	Cell viability %	Mean %
*A. paniculata* 1	86%	86%
*A. paniculata* 2	88%	
*A. paniculata* 3	82%	
*A. paniculata* 4	90%	
*A. paniculata* 5	84%	

### Estimation of total carbohydrate content by phenol sulphuric acid method

To determine the dose concentration of each elicitor, Phenol sulphuric acid method was used to find out the total carbohydrate content present in each elicitor which revealed 9.8 mg, 14.7 mg and 19.66 mg of fungal polysaccharide l^-1^ in 1.0 ml, 1.5 ml and 2.0 ml of *Aspergillus niger* elicitor respectively. Similarly, 0.3%, 0.6% and 1.2% of the homogenate of *Penicillium expansum* were equivalent to 4.08 mg, 8.16 mg and16.32 mg of fungal polysaccharide l^-1^ respectively.

## HPLC result

**Figure 2:**
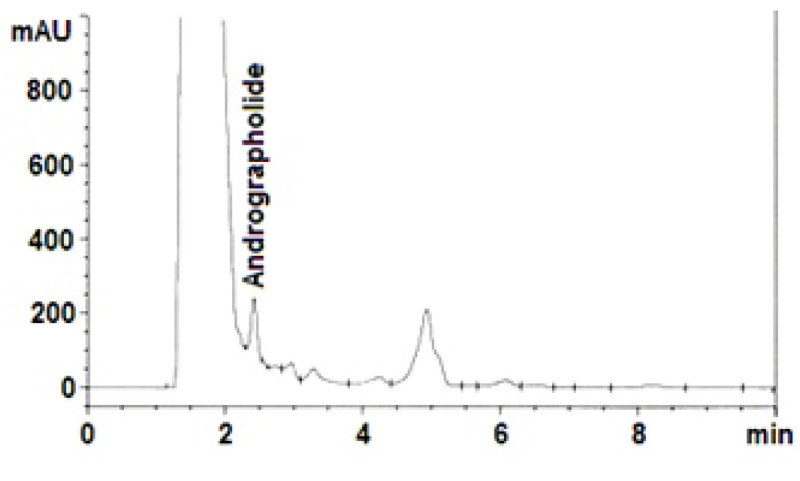
**HPLC chromatogram showing the chromatographic separation of andrographolide from other compounds of the fungal elicited cell suspension cultures extract of** ***A. paniculata.***

A simple, isocratic, selective reversed phase-liquid chromatographic method was developed for the determination of andrographolide in the extracts of the cell suspension cultures of *Andrographis paniculata* (Burm. f.) Nees. In this method, a satisfactory separation was obtained with a mobile phase consisting of Acetonitrile: 0.1% (v/v) phosphoric acid in water (40: 60, v/v). Quantification was achieved with UV detection at 223 nm based on peak area (Figure 2). The average retention time with ± standard deviation was found to be 2.41 ± 0.05 min and 2.33 ± 0.017 min for andrographolide in *Aspergillus niger* and *Penicillium expansum* treated samples, respectively.

### Effect of *Aspergillus niger* elicitors on quantitative enhancement in andrographolide content

**Figure 3:**
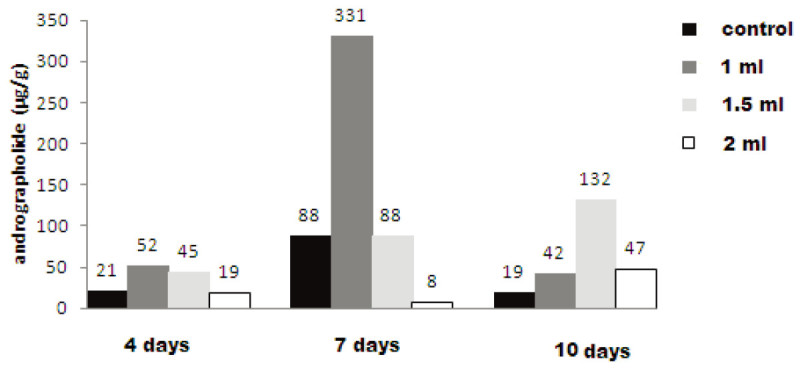
**Effect of** ***Aspergillus niger*** **on andrographolide synthesis in** ***A. paniculata*** **cell suspension culture.**

Figure 3 display the elicitation of andrographolide by *Aspergillus niger* elicitors in cell suspension culture of *Andrographis paniculata*. In 4 days and 7 days treatment duration, 1 ml of *A. niger* extract was found to be most positive concentration for eliciting andrographolide compound. The estimated quantity of andrographolide was 52.0 μg/g and 331.0 μg/g in 4 days and 7 days treatment duration, respectively, which showed 2.47 and 3.76 fold increase over their respective controls.

Increasing doses of *A. niger* extract with 4 days treatment duration had moderate effect on andrographolide content compared to respective control (Table 2). The concentration of 1.5 ml of *A. niger* extract produced 45.0 μg/g of andrographolide content which was slightly higher over the control and 2 ml showed 19.0 μg/g of andrographolide, a less quantity compared to respective control (21.0 μg/g). For 7 days treatment duration, only 1 ml dose was found to be effective in eliciting andrographolide content (331.0 μg/g) whereas, 1.5 ml concentration revealed negative response. Two ml extract treatment indicates negligible quantity of andrographolide (8.0 μg/g) i.e. lower than control (88.0 μg/g).

**Table 2:** **Effect of fungal elicitors on andrographolide production (Mean ± SD)**

Name of elicitors	Treatment durations	Concentrations of elicitor	Andrographolide content in μg/g	Fold increase
		Control	21.0 ± 0.74	
*Aspergillus niger*	4 days	1.0 ml	52.0 ± 0.13	2.47
		1.5 ml	45.0 ± 0.61	2.14
		2.0 ml	19.0 ± 0.3	-
		Control	88.0 ± 0.37	
	7 days	1.0 ml	331.0 ± 0.43	3.76
		1.5 ml	88.0 ± 0.34	-
		2.0 ml	8.0 ± 0.70	-
		Control	19.0 ± 0.13	
	10 days	1.0 ml	42.0 ± 0.94	2.21
		1.5 ml	132.0 ± 0.62	6.94
		2.0 ml	47.0 ± 0.74	2.47
		Control	10.0 ± 0.11	
*Penicillium expansum*	2 days	0.3%	12.0 ± 0.84	1.2
		0.6%	22.0 ± 0.83	2.2
		1.2%	16.0 ± 0.69	1.6
		Cont.	24.0 ± 0.28	
	5 days	0.3%	22.0 ± 0.86	-
		0.6%	35.0 ± 0.95	1.45
		1.2%	32.0 ± 0.11	1.33
		Cont.	13.0 ± 0.45	
	8 days	0.3%	29.0 ± 0.14	2.23
		0.6%	81.0 ± 0.74	6.23
		1.2%	15.0 ± 0.21	1.15

The 10 days treatment duration proved to be most favorable as it indicates enhancement in the content of andrographolide in 1 ml of *A. niger* extract. Increased concentration of *A. niger* extract (1.5 ml), revealed remarkable increase in andrographolide content (132.0 μg/g). However, further increase in the concentration to 2 ml, ceased the production of andrographolide to 47.0 μg/g. The results obtained in the present study are in agreement with the findings of other researchers which suggest that the lower dose of *A. niger* extract is effective in eliciting the target compound (Ibrahim et al.^2007^; Fu and Lu^1999^). It is reported that 2.5 ml of fungal extract prepared from fungal strain (F5) which was isolated from inner bark of 15-20-m high *Taxus chinensis* tree showed enhanced paclitaxel content on 14 days of treatment exposure in *T. chinensis* cell suspension culture (Zhang et al.^2000^). Similarly, 2 ml extract of *A. niger* per 100 ml of cell suspension culture enhanced oleandrin yield up to 3.164 mg l^-1^. It was 8.8 fold higher than that of control (0.31 mg l^-1^) in 25 days old culture of *Nerium oleander* (Ibrahim et al.^2007^).

### Effect of *Penicillium expansum* elicitors on quantitative enhancement in andrographolide content

**Figure 4:**
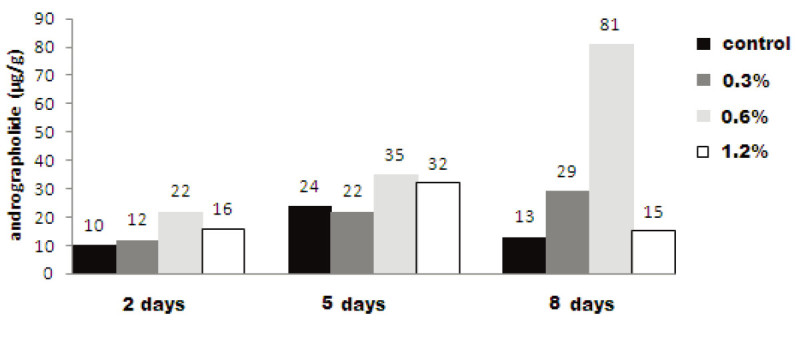
**Effect of** ***Penicillium expansum*** **on andrographolide synthesis in** ***A. paniculata*** **cell suspension culture.**

Table 2 and Figure 4 illustrate the effect of different concentrations of *P. expansum* on andrographolide production for different treatment durations in cell suspension culture of *Andrographis paniculata*. In 2 and 5 days treatment duration, 0.6% *P. expansum* extract was found most suitable concentration for andrographolide elicitation which yielded 22.0 μg/g and 35.0 μg/g of andrographolide respectively. These enhancements were 2.2 and 1.45 fold higher than their respective controls. Andrographolide yield enhanced maximum as 81.0 μg/g, when treated with 0.6% *P. expansum* extract for 8 days. It was 6.23 fold higher than the control which reached up to 13.0 μg/g for the same culture period.

Similar results have been obtained in *Beta vulgaris* hairy root cultures in which the lower concentrations of *Penicillium notatum* (5% and 7%) have yielded 40.5 mg/l and 46.2 mg/l of betalain respectively compared to control 24.5 mg/l when exposed to 3 days incubation period (Savitha et al.^2006^). The further enhancement was achieved up to 92.0 mg/l and 97.2 mg/l of betalain in 5% and 7% concentration of *Penicillium notatum* elicitor treated samples on 7 days treatment period over control (43.2 mg/l).

**Figure 5:**
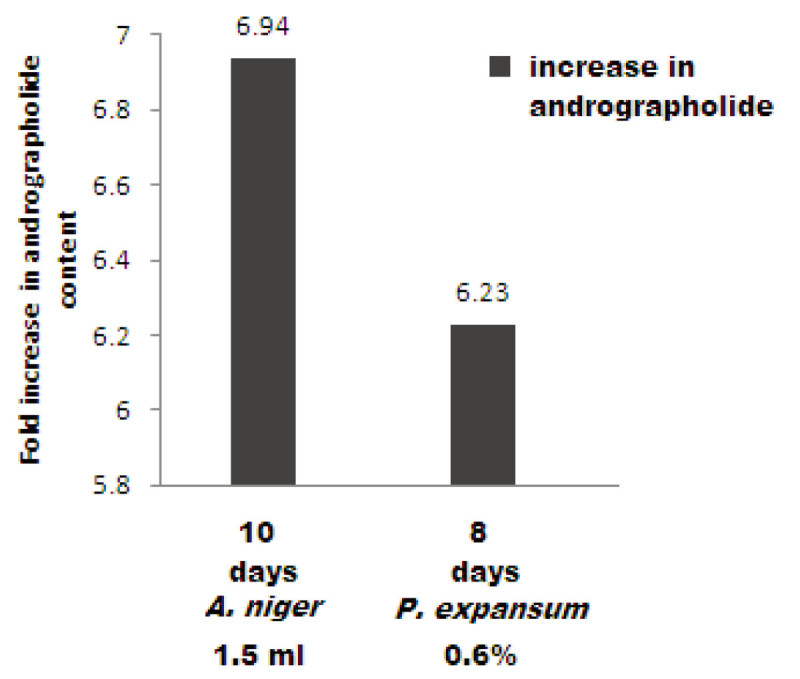
**Optimal fold increase in andrographolide.**

*Aspergillus niger* elicitor (1.5 ml, 10 days duration) showed 6.94 fold enhancement in andrographolide content which was higher among other studied concentrations and treatment durations of *A. niger. P. expansum* elicitor showed 6.23 fold enhancements in andrographolide content when 0.6% concentration of *P. expansum* for 8 days treatment duration was administered. This concentration and incubation period was superior to other studied concentrations and incubation periods for *P. expansum* elicitor. The elicitation effect of *A.* niger was higher (6.94 fold increase in andrographolide content) compared to *P. expansum* (6.23 fold increase) in *A. paniculata* cell suspension culture when treated with 1.5 ml of *A.* niger extract for 10 days and 0.6% concentration of *P. expansum* for 8 days treatment duration respectively (Figure 5).

Plant cells are interacted with elicitors at metabolic level. Plants on pathogen attack or treatment of plants with elicitor causes an array of defense reaction which includes the accumulation of a wide range of secondary metabolites at enhanced level. Various mechanisms have been hypothesized for mechanism of elicitation. The binding of elicitor to a plasma membrane receptor is hypothesized by some researcher for elicitation process (Cheong and Hahn^1991^; Cosio et al.^1990^; Basse et al.^1993^; Nürnberger et al.^1994^; Braun and Walker^1996^and Hanania and Avni^1997^). During the process of elicitation, elicitors are recognized by specific receptors in the plant cell which are localized either on the cell surface for a number of fungal elicitors or within the cell for certain bacterial elicitors (Ebel and Scheel^1997^; Yang and Gabriel^1995^and Ackerveken et al.^1996^). These receptors initiate signaling processes that activate plant defenses. The pathogen-plant interaction can be divided into two different categories viz. host or race-specific resistance and non-host interaction. In case of the host or race-specific resistance, the pathogen interacts with a potential host that has acquired a resistance gene (R-gene). In case of the non-host interaction, the plant recognizes potentially pathogenic microbes by the means of conserved structures which are present in a wide variety of microbes. These structures are known as pathogen-associated molecular patterns (PAMP).

Many elicitors such as chitin, xyloglucans, chitosan, h-glucan and oligogalacturonide are known to induce phytoalexins in different plant systems, suggesting that different plants possess some common receptors to sense these signals. According to Gelli et al. (1997), Ca^2+^ influx into the cytoplasm from extracellular environment and intracellular Ca^2+^ reservoir is involved in elicitation process. For example, in parsley cells, an elicitor- responsive calcium channel has been identified and characterized and a transient influx of calcium has been found after the addition of fungal elicitor (Zimmermann et al.^1997^). Some researchers have reported mitogen-activated protein kinase (MAPK) and G-protein activation responsible for elicitation. (Droillard et al.^2000^; Agrawal et al.^2002^).

Changes in protein phosphorylation pattern and protein kinase activation on elicitation have been reported during elicitation process (Felix et al.^1991^; Yang et al.^1997^; Romeis^2001^and Roos et al.^1999^). Armero and Tena (2001) have suggested that acidification of cytoplasm caused by H^+^-ATPase inactivation could be involved in elicitation process. In elicitor treated plant tissues, decrease in membrane polarization and increase in extracellular pH has been considered for elicitation process (Pugin et al.^1997^; Bolwell et al.^1995^). Apostol et al. (1989) suggested that the production of ROS such as superoxide anion and H_2_O_2_ are responsible for elicitation process. This report has been supported by Low and Merida (1996) who observed that involvement of ROS in cross linking of cell wall bound protein rich components can act as a secondary messenger and it is involved in activation of defense genes.

The demand of natural product is increasing proportionally due to medico clinical applications of plant products. However, the natural plant resources day by day are either in the threat or depleting. As a consequence, there is a possibility of creating a big vacuum with respect to the natural product produced and its increasing demands. Such situation reflects adversely two ways, the inadequate availability of essential bioactive plant product and the inflation in their cost. As a consequence, the utility of a particular product to the society is adversely affected and hence, the serious attentions are required to be paid by initiating the innovations and research in plant science that will contribute to fill up the lacunae to some extent if created in the future. The concept of secondary metabolite engineering is therefore, gained high importance since, it targets straight to the bioactive compounds. The present experimental work is a small effort towards this direction. The experimental system, *Andrographis paniculata* is cultivated in Ayurvedic nurseries due to its medicinal importance. When the supply of this particular plant resource is declined, the qualitative and quantitative enhancement of metabolite products by in vitro techniques remains the only way to obtain a desirable plant product.

## Conclusion

The treatment duration is reported as an important criterion for the elicitation mechanism besides selection of suitable concentration of selected elicitors. Both the fungal elicitors (*A. niger* and *P. expansum*) showed that longer treatment duration is suitable for eliciting andrographolide synthesis. This may be attributed to the presence of polysaccharides in the fungal extract for eliciting andrographolide when applied in moderate quantity.

## Authors’ original submitted files for images

Below are the links to the authors’ original submitted files for images. Authors’ original file for figure 1 Authors’ original file for figure 2 Authors’ original file for figure 3 Authors’ original file for figure 4 Authors’ original file for figure 5

## Abbreviations

PAMP: Pathogen-associated molecular patterns
ROS: Reactive oxygen species
PDA: Potato dextrose agar
BAP: Benzyl amino purine.

## References

[CR1] Ackerveken VG, Marois E, Bonas U (1996). Recognition of the bacterial avirulence protein AvrBs3 occurs insde the host plant cell. Cell.

[CR2] Agrawal GK, Rakwal R, Iwahashi H (2002). Isolation of novel rice (*Oryza sativa* L.) multiple stress responsive MAP kinase gene, OsMSRMK2, whose mRNA accumulates rapidly in response to environmental cues. Biochem. Biophys Res. Commun.

[CR3] Apostol L, Heinstein PF, Low PS (1989). Rapid stimulation of an oxidative burst during elicitation of cultured plant cells. Plant Physiol.

[CR4] Armero J, Tena M (2001). Possible role of plasma membrane H^+^-ATPase in the elicitation of phytoalexin and related isoflavone root secretion in chickpea (*Cicer arietinum* L.) seedlings. Plant Sci.

[CR5] Basse CW, Fath A, Boller T (1993). High affinity binding of a glycopeptide elicitor to tomato cells and microsomal membranes and displacement by specific glycan suppressors. J. Biol. Chem.

[CR6] Bojwani SS, Razdan MK (1996). Plant tissue culture: theory and practice, a revised edition.

[CR7] Bolwell GP, Buti VS, Davies DR, Zimmerlin A (1995). The origin of the oxidative burst in plants. Free Radical Res.

[CR8] Braun DM, Walker JC (1996). Plant transmembrane receptors: new pieces in the signaling puzzle, Trends Biochem. Sci.

[CR9] Brodelius P, Funk C, Haner A, Villegas M (1989). A procedure for the determination of optimal chitosan concentrations for elicitation of cultured plant cells. Phytochemistry.

[CR10] Calabrese C, Berman SH, Babish JG, Ma X, Shinto L, Dorr M, Wells K, Wenner CA, Standish LJ (2000). A phase I trial of andrographolide in HIV positive patients and normal volunteers. Bastyr University Research Institute, Bastyr University, Washington 98028, USA. Phytother Res.

[CR11] Cheong JJ, Hahn MG (1991). A specific, high-affinity binding site for the heptaglucoside elicitor exists in soybean membranes. Plant Cell.

[CR12] Cosio EG, Frey T, Verduyn R, Boom JV, Ebel J (1990). High affinity binding of a synthetic heptaglucoside and fungal glucan phytoalexin elicitors to soybean membranes. FEBS Lett.

[CR13] Droillard MJ, Thibivilliers S, Cazale AC, Barbier BH, Lauriere C (2000). Protein kinases induced by osmotic stresses and elicitor molecules in tobacco cell suspensions: two crossroad MAP kinases and one osmoregulation-specific protein kinase. FEBS Lett.

[CR14] Ebel J, Scheel D, Caroll GC, Tudzynski P (1997). The Mycota V, part A. Plant relationships.

[CR15] Felix G, Grosskopf DG, Regenass M, Boller T (1991). Rapid changes of protein phosphorylation are involved in transduction of the elicitor signal in plant cells. Proc. Natl. Acad. Sci. USA.

[CR16] Fu XQ, Lu DW (1999). Stimulation of shikonin production by combined fungal elicitation and *in situ* extraction in suspension cultures of *Arnebia euchroma*. Enzyme Microbial Technol.

[CR17] Gelli A, Higgins VJ, Blumwald E (1997). Activation of plant plasma membrane Ca^2+^-permeable channels by race specific fungal elicitors. Plant Physiol.

[CR18] Guava ViaCount Reagent (2006). Guava technologies, 4600–0010 rev c.

[CR19] Hanania U, Avni A (1997). High-affinity binding site for ethylene-inducing xylanase elicitor on *Nicotiana tabacum* membranes. Plant J.

[CR20] Ibrahim AK, Khalifa S, Khafagi I, Youssef D, Khan I, Mesbah M (2007). Stimulation of oleandrin production by combined *Agrobacterium tumefaciens* mediated transformation and fungal elicitation in *Nerium oleander* cell cultures. Enzyme and Microbial Technology.

[CR21] Kumar RA, Sridevi K, Kumar NV, Nanduri S, Rajagopal S (2004). Anticancer and immunostimulatory compounds from *Andrographis paniculata*. J Ethnopharmacol.

[CR22] Low PS, Merida JR (1996). The oxidative burst in plant defense: function and signal transduction. Physiol. Plant.

[CR23] Masanaru M (1994). Plant tissue culture an alternative for production of useful metabolites.

[CR24] Mendhulkar VD, Ali Moinuddin M, Raut RW (2009). Saponin estimation in *Vigna radiata* cell culture treated with cell permeabilizing agent. Triton X-100. Advances in Plant Sciences.

[CR25] Murashige T, Skoog F (1962). A revised medium for rapid growth and bioassays with tobacco tissue cultures. Physiol. Plant.

[CR26] Nürnberger T, Nennstiel D, Jabs T, Sacks WR, Hahlbrock K, Scheel D (1994). High affinity binding of a fungal oligopeptide elicitor to parsley plasma membranes triggers multiple defense responses. Cell.

[CR27] Pugin A, Frachisse JM, Tavernier E, Bligny R, Gout E, Douce R, Guern J (1997). Early events induced by the elicitor cryptogein in tobacco cells: involvement of a plasma membrane NADPH oxidase and activation of glycolysis and the pentose phosphate pathway. The Plant Cell.

[CR28] Purohit SS, Vyas SP (2004). Medicinal plant cultivation –a scientific approach. (Agrobios-India, publ.).

[CR29] Raskin I, Ribnicky DM, Komarnytsky S, Ilic N, Poulev A, Borisjuk N, Brinker A, Moreno DA, Ripoll C, Yakoby N, O’Neal JM, Cornwell T, Pastor I, Fridlender B (2002). Plants and human health in the twenty-first century. Trends Biotechnol.

[CR30] Rates SMK (2001). Plants as sources of drugs. Toxicon.

[CR31] Romeis T (2001). Protein kinases in the plant defense response. Curr Opin Plant Biol.

[CR32] Roos W, Dordschbal B, Steighardt J, Hieke M, Weiss D, Saalbach G (1999). A redoxdependent, G-protein-coupled phospholipase A of the plasma membrane is involved in the elicitation of alkaloid biosynthesis in Eschscholtzia californica. Biochim Biophys Acta.

[CR33] Sadasivam S, Manickam A (2005). Biochemical methods.

[CR34] Savitha BC, Thimmaraju R, Bhagyalakshmi N, Ravishankar GA (2006). Different biotic and abiotic elicitors influence betalain production in hairy root cultures of *Beta vulgaris* in shake-flask and bioreactor. Process Biochemistry.

[CR35] Vanisree M, Lee CY, Lo SF, Nalawade SM, Lin CY, Tsay HS (2004). Studies on the production of some important secondary metabolites from medicinal plants by plant tissue culture. Bot. Bull. Acad. Sin.

[CR36] Yang Y, Gabriel DW (1995). Xanthomonas avirulence/pathogenicity gene family encodes functional plant nuclear targeting signals. Mol. Plant Microbe Interact.

[CR37] Yang J, Yu M, January YN, January LY (1997). Stabilization of ion selectivity alters by pore loop ion pairs in an inwardly rectifying potassium channel. Proc. Natl. Acad. Sci. USA.

[CR38] Zhang CH, Mei XG, Liu L, Yu LJ (2000). Enhanced paclitaxel production induced by the combination of elicitors in cell suspension cultures of *Taxus chinensis*. Biotechnology Letters.

[CR39] Zhao HY, Frang WY (1991). Antithrombotic effect of *Andrographis paniculata* Nees in preventing myocardial infarction. Chi. Med. J. (Engl).

[CR40] Zimmermann S, Nürnberger T, Frachisse JM, Wirtz W, Guern J, Hedrich R, Scheel D (1997). Receptor-mediated activation of a plant Ca21-permeable ion channel involved in pathogen defense. Proc. Natl. Acad. Sci. USA.

