# Supplementary figures and images for: Enhanced synthesis of andrographolide by Aspergillus niger and Penicillium expansum elicitors in cell suspension culture of Andrographis paniculata (Burm. f.) Nees
Source: Bot Stud. 2013 Oct 24;54:49. doi: 10.1186/1999-3110-54-49 (PMC5430361; doi:10.1186/1999-3110-54-49)

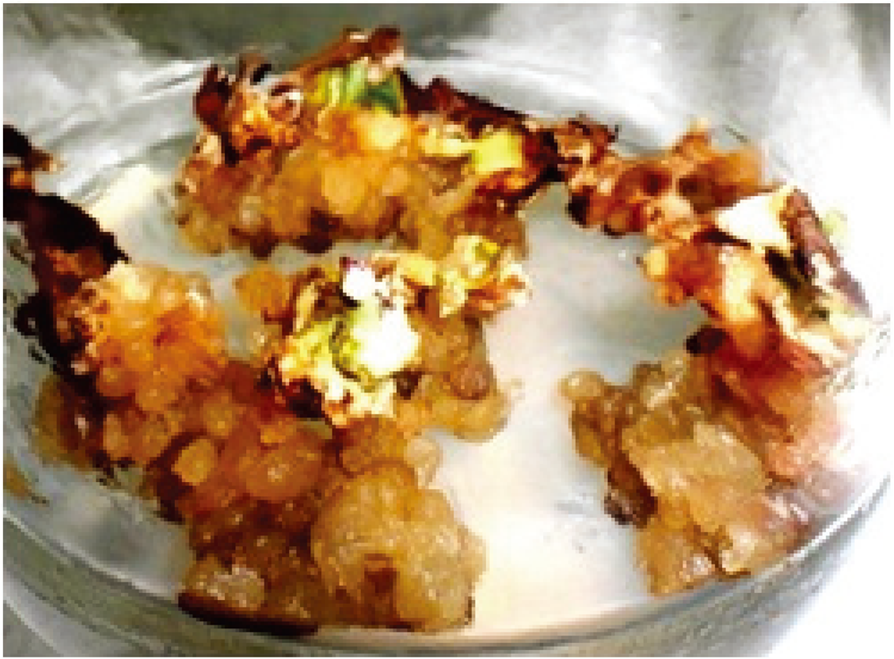

Supplement: Supplementary file 1 — Authors’ original file for figure 1 [file 40529_2013_43_MOESM1_ESM.tif]

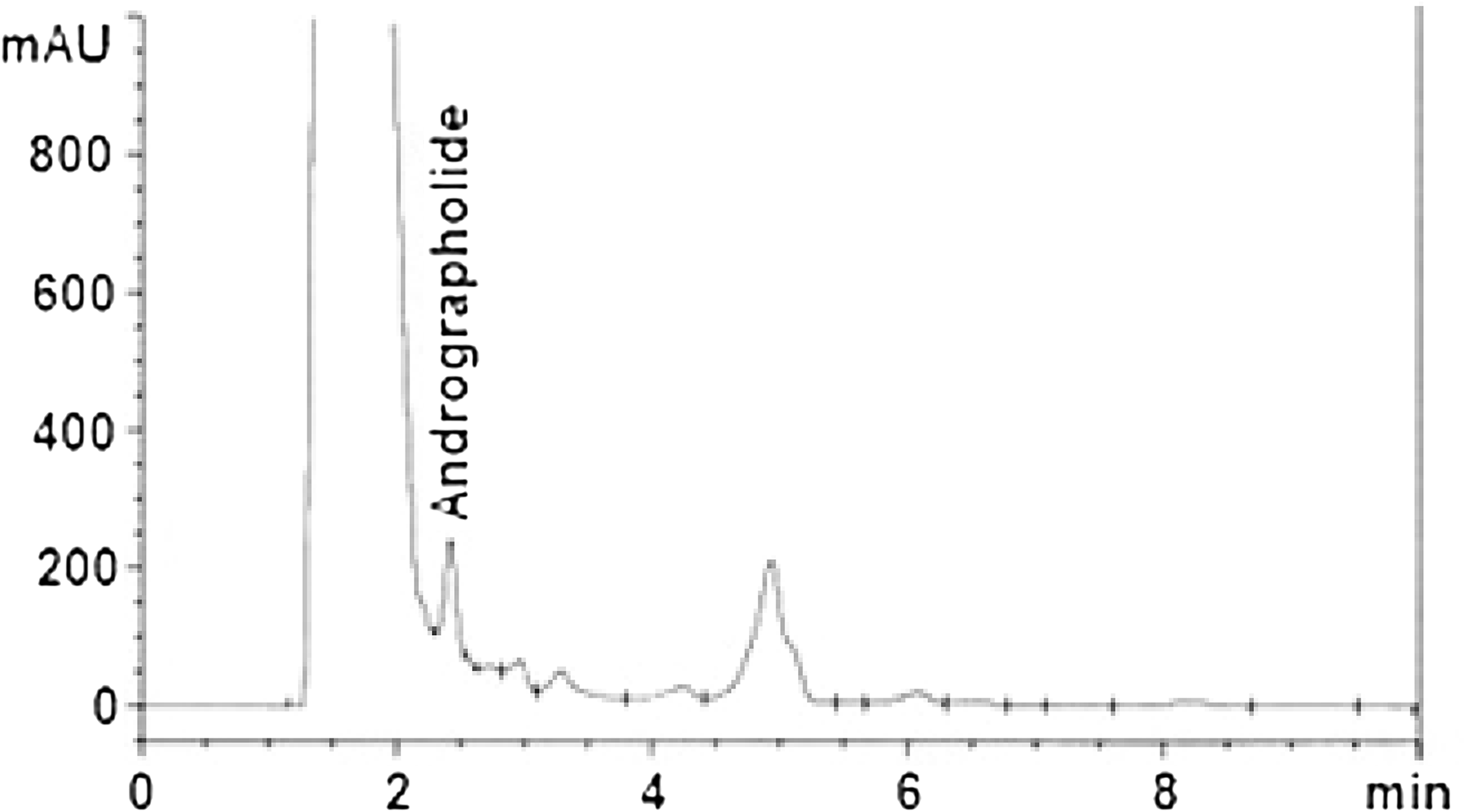

Supplement: Supplementary file 2 — Authors’ original file for figure 2 [file 40529_2013_43_MOESM2_ESM.tif]

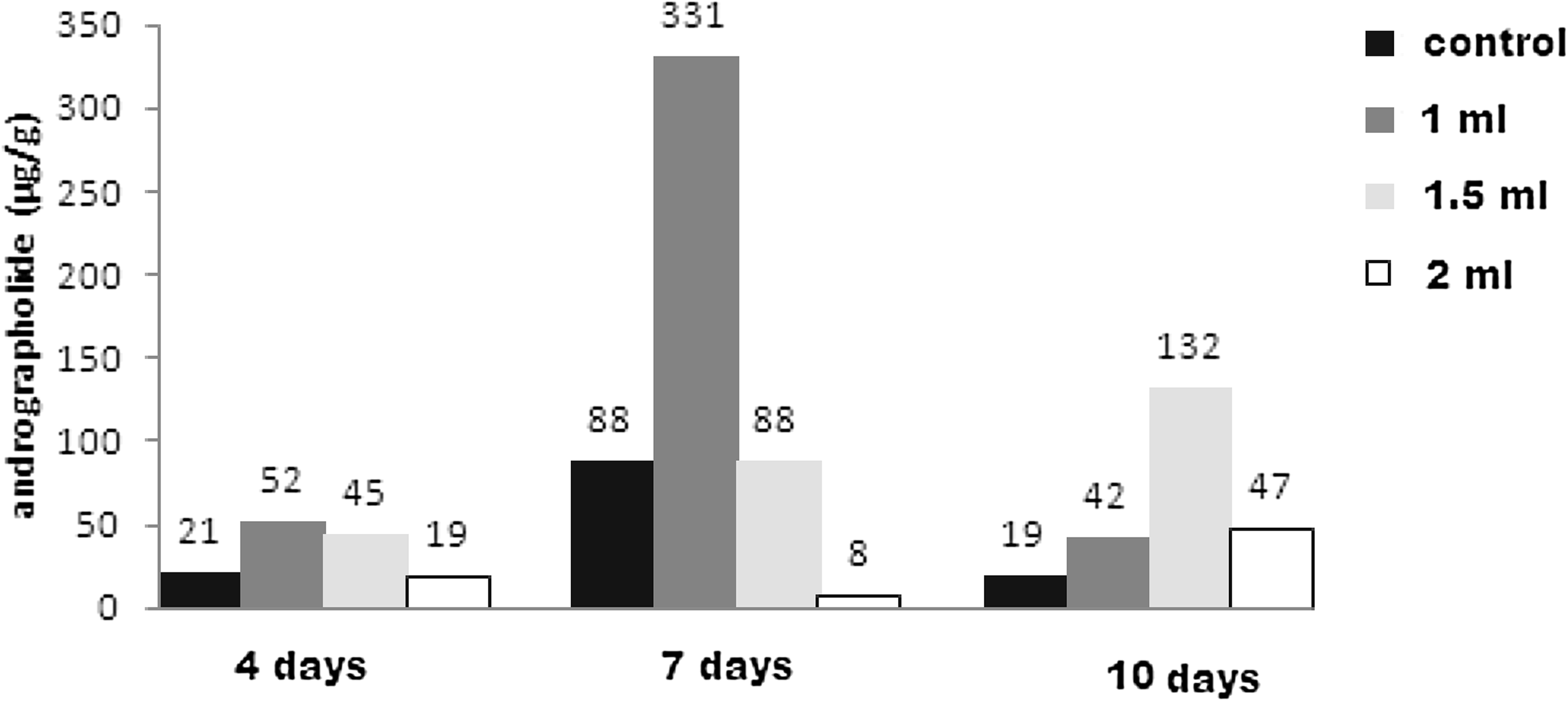

Supplement: Supplementary file 3 — Authors’ original file for figure 3 [file 40529_2013_43_MOESM3_ESM.tif]

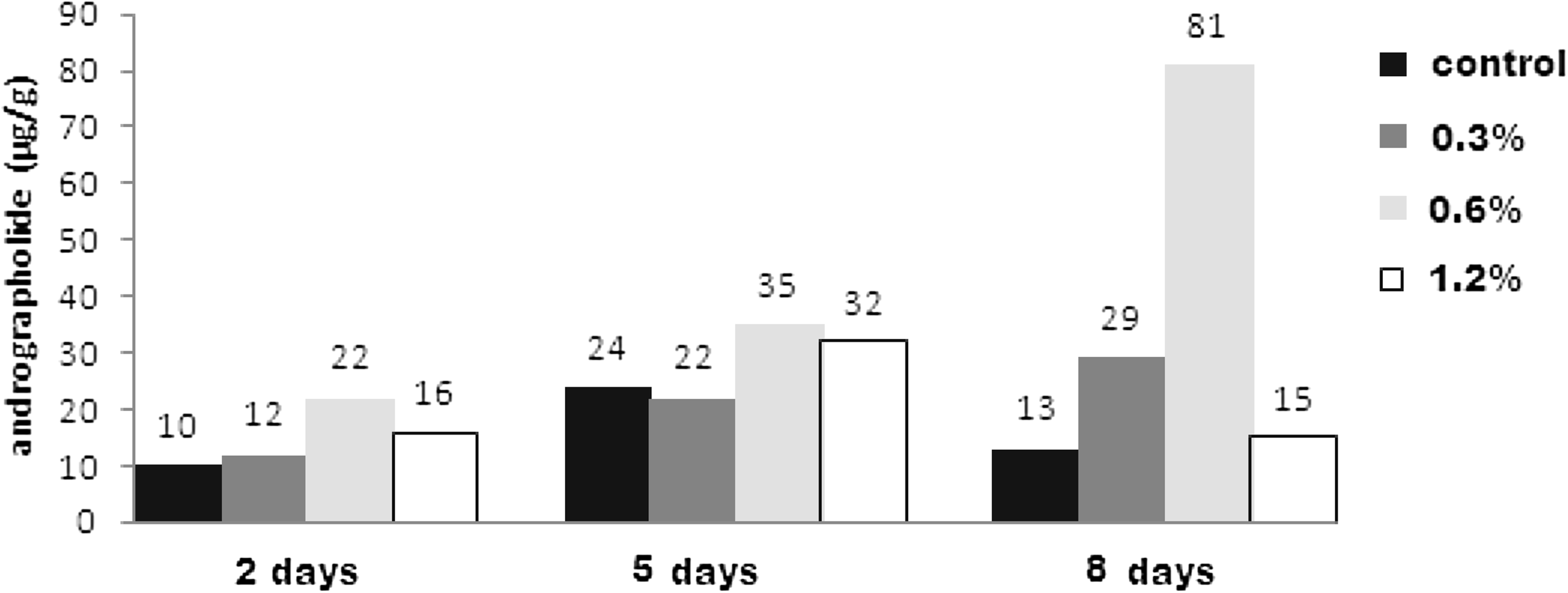

Supplement: Supplementary file 4 — Authors’ original file for figure 4 [file 40529_2013_43_MOESM4_ESM.tif]

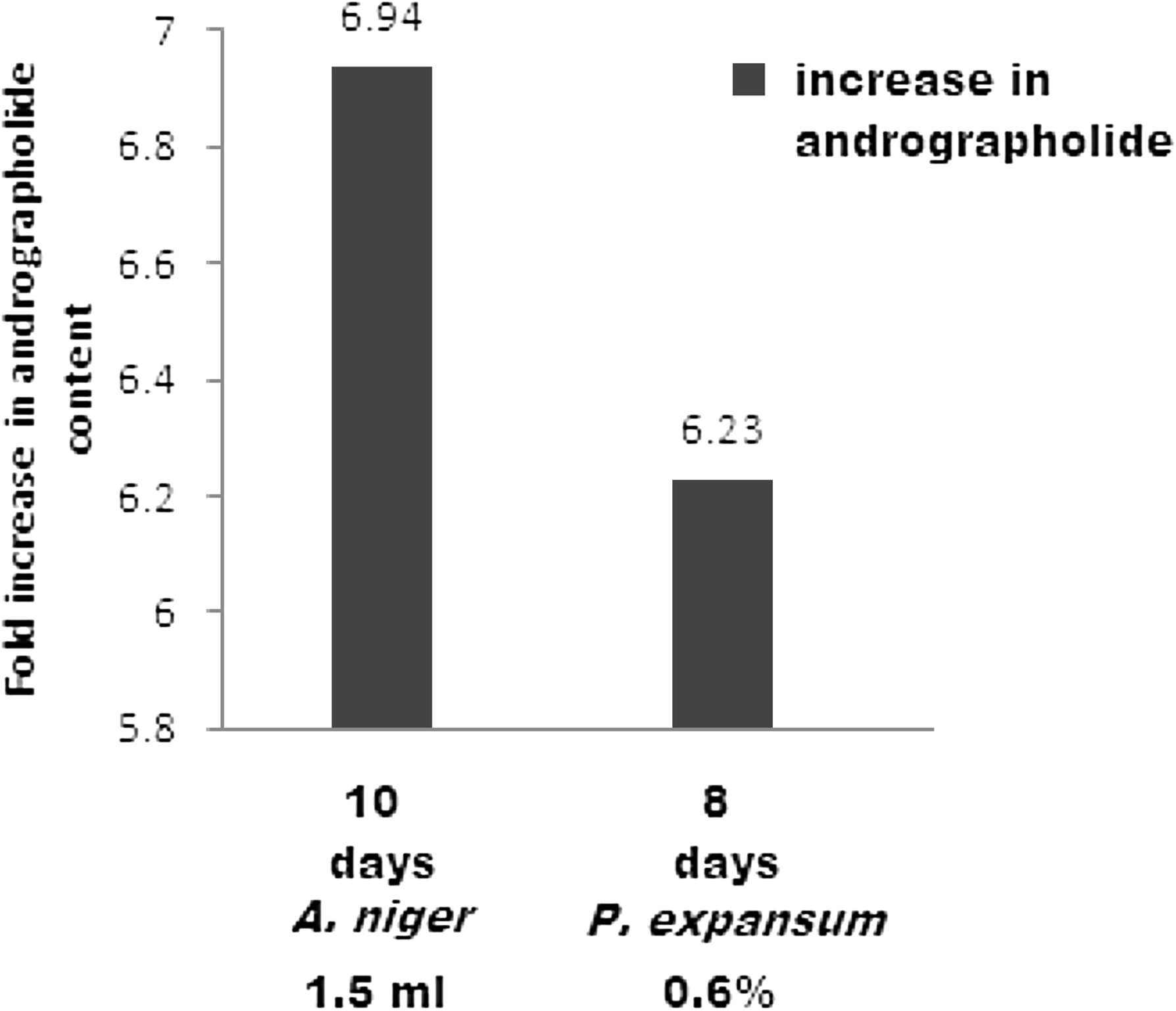

Supplement: Supplementary file 5 — Authors’ original file for figure 5 [file 40529_2013_43_MOESM5_ESM.tif]
